# Consideration of serum IL‐36α and β levels trends in two patients with chikungunya fever

**DOI:** 10.1002/ccr3.7680

**Published:** 2023-07-17

**Authors:** Makoto Kondo, Yoshiaki Matsushima, Takehisa Nakanishi, Shohei Iida, Koji Habe, Keiichi Yamanaka

**Affiliations:** ^1^ Department of Dermatology, Graduate School of Medicine Mie University Tsu Japan

**Keywords:** chikungunya fever, IL‐36, IL‐36α, IL‐36β, immune defense, joint pain

## Abstract

**Key Clinical Message:**

IL‐36 might play a role as an initial immune mechanism against chikungunya fever, and regulating IL‐36 production could be a potential treatment approach for this condition.

**Abstract:**

Two Japanese siblings visited Cook Islands in 2015 and developed Chikungunya fever upon their return. The sister experienced high fever, joint pain, and leg swelling, while the brother had joint pain and a rash. Both siblings had a confirmed CHIKV infection and continued to experience prolonged joint pain, with the sister enduring chronic pain for about a year. In this study, the levels of IL‐36 in the serum of two siblings who were infected with chikungunya fever during the acute and recovery phases were compared using ELISA. IL‐36 is a cytokine that induces inflammation and is produced by cells in tissues such as the skin and mucosa. It was hypothesized that IL‐36 may be involved in persistent joint pain after chikungunya fever infection. Both siblings experienced long‐lasting joint pain after chikungunya fever infection. The levels of IL‐36α and IL‐36β decreased by 56 days after infection. In the results, IL‐36 plays an important role in host immunity and may act as part of the immune response during chikungunya virus infection. Inhibiting the release of IL‐36 could be a promising approach for developing new treatment methods for chikungunya fever.

## 1 INTRODUCTION

Chikungunya fever is a persistent joint pain disease caused by mosquito‐borne arboviruses: chikungunya virus (CHIKV). The symptoms of chikungunya fever include high fever, joint pain, muscle pain, headache, rash, nausea, and fatigue.^1^ While most people recover completely within a few weeks, symptoms can also persist for a prolonged period. However, some patients who have been infected chikungunya fever continue to experience persistent joint pain even after recovering.^2^ Interleukin (IL)‐6 and IL‐17 has been reported to be a key cytokine in persistent joint pain following chikungunya fever infection.^3^, ^4^, ^5^ Recently, it has been reported that IL‐36 is involved in persistent joint pain, such as in psoriatic arthritis.^6^ Therefore, we hypothesized that IL‐36 may also be involved in persistent joint pain after chikungunya fever infection. In addition, IL‐36 is a type of cytokine that induces inflammation and is produced by cells such as epithelial cells, fibroblasts, and dendritic cells in tissues such as the skin and mucosa.^7^ It is primarily expressed in epithelial cells and is known to induce immune responses and inflammatory reactions against infections such as bacteria and fungi.

In this study, we compared the levels of IL‐36 using ELISA in the serum of siblings who were infected with Chikungunya fever during the acute and recovery phases of the infection. Our report investigates how IL‐36 is involved in chikungunya fever. Understanding whether IL‐36 plays a role in persistent joint pain or in the immune defense mechanisms during chikungunya fever infection could lead to the development of new treatment strategies.

## 2 CASE PRESENTATION

We previously reported two cases with chikungunya fever after traveling to Cook Island.^8^ The abstract of this case is as follows: In the winter of 2015, a brother and sister from Japan visited the Cook Islands. Upon their return to Japan, the sister developed a high fever, joint pain, and leg swelling, while the brother experienced joint pain and a widespread rash. Initial tests for dengue fever were negative, but the sister tested positive for CHIKV. Further tests confirmed a past infection with CHIKV in both siblings. They continued to experience long‐lasting joint pain. Especially, the sister has been suffering from chronic joint pain for about 1 year.

**FIGURE 1:**
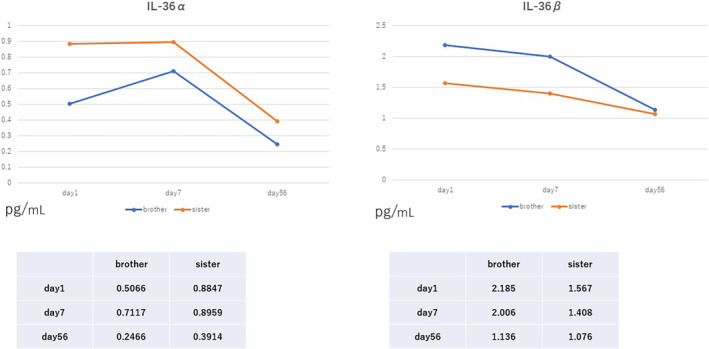
The graphs show the changes in serum levels of IL‐36α and IL‐36β measured on Day 1 (at first visit), Day 7, and Day 56 from the brother and sister. A 20‐fold dilution serum was used for the assay.

In recent years, cytokines that contribute to joint pain associated with various diseases have been identified. As CHIKV infection, elevated serum IL‐6, IL‐17, IL‐21, and IL‐23 levels have been reported in patients with chronic arthritis after chikungunya fever. In addition, recent findings in psoriatic arthritis revealed the elevated serum IL‐36 during arthritis. IL‐36 is a relatively newly found cytokine belonging to the IL‐1 family, whose involvement in various diseases is still poorly understood. We thought that if we could clarify how IL‐36 is involved in the pathogenesis and joint symptoms of chikungunya fever, it might lead to the prevention and treatment of the disease. In the current study, to evaluate the involvement of IL‐36 in patients with chikungunya fever, stocked serum from the previously reported two cases at the initial visit, 7 days, and 56 days later were used to measure IL‐36α and IL‐36βconcentration. IL‐36α and IL‐36β were measured in the patient's serum using human IL‐36α/IL‐1F6 and IL‐36β/IL‐1F8 DuoSet ELISA® (R&D Systems). At the initial visit, IL‐36α was higher in the sister, who had more fever and arthralgia symptoms than her brother. Seven days later, both had higher levels than at the initial visit, but the brother, whose symptoms of Chikungunya fever appeared later, had a larger increase than at the initial visit. After 56 days, both decreased to about half of the peak value. IL‐36β was highest at the first visit for both patients and gradually decreased (Figure 1).

## 3 DISCUSSION

Chikungunya fever causes arthritis, which often persists for a long time in many cases. The sibling patients also suffered from arthralgia even with taking non‐steroidal anti‐inflammatory drugs. The sister in particular had severe symptoms of arthralgia that persisted for about a year. Therefore, as reported in recent years, we focused on the association between IL‐36 and joint pain. Consequently, we measured the fluctuation of IL‐36 levels in two patients who had contracted chikungunya fever. These two patients were infected at the same time and place and were siblings, which made them less susceptible to genetic and racial background influences and more suitable for unbiased evaluation. In addition, based on the serum IL‐36α and IL‐36β levels results, the association between IL‐36 and chikungunya fever was discussed. Both IL‐36α and IL‐36β were reduced by 56 days. In the case of psoriatic arthritis, IL‐36 activates dendritic cells inducing Th17 cells to generate IL‐17 and IL‐22, resulting in persistent arthritis. Therefore, the mechanism of chronic arthritis caused by psoriatic arthritis is different from that of chronic arthritis in chikungunya fever and IL‐36 may not be involved in the chronic arthritis of chikungunya fever. IL‐36α is produced by monocytes, B cells, and T cells. IL‐36β is produced by dendritic cells and induces proinflammatory cytokines, including IL‐12, IL‐1β, IL‐6, and TNF‐α. Both IL‐36α and IL‐36β play an important role in host immunity, and it has been suggested that one physiological function of IL‐36 may be to counteract microbial immune evasion.^9^ IL‐36β levels were highest initially, indicating induced inflammatory cytokines during infection control, while IL‐36α remained persistently high or increased, suggesting peak acquired immune response. IL‐36 potentially acts as an immunological defense mechanism during chikungunya virus infection.

## 4 CONCLUSION

Currently, the treatment for chikungunya fever is primarily focused on managing the symptoms, as no specific licensed vaccines, antibodies for immunotherapy, or antiviral drugs are available for this infection. The main approach is to provide symptomatic relief, such as pain and fever management, hydration, and rest. Based on these findings, it is reasonable to consider that inhibiting the release of IL‐36 could be a promising approach for developing new treatment methods for chikungunya fever.

## AUTHOR CONTRIBUTIONS

**Makoto Kondo:** Conceptualization; data curation; formal analysis; investigation; writing – original draft. **Yoshiaki Matsushima:** Data curation. **Takehisa Nakanishi:** Data curation; formal analysis. **Shohei Iida:** Data curation. **Koji Habe:** Writing – review and editing. **Keiichi Yamanaka:** Project administration; writing – review and editing.

## FUNDING INFORMATION

The authors did not receive any financial support for this study.

## CONFLICT OF INTEREST STATEMENT

The authors have declared that no competing interests exist.

## CONSENT STATEMENT

Written informed consent was obtained from the patient to publish this report in accordance with the journal's patient consent policy.

## Data Availability

The original contributions presented in this study are included in the article. Further inquiries can be directed to the corresponding authors.
